# Assessing inter- and intra-rater reliability of movement scores and the effects of body-shape using a custom visualisation tool: an exploratory study

**DOI:** 10.1186/s13102-024-00988-1

**Published:** 2024-09-30

**Authors:** Gwyneth B. Ross, Xiong Zhao, Nikolaus F. Troje, Steven L. Fischer, Ryan B. Graham

**Affiliations:** 1 School of Human Kinetics, Faculty of Health Sciences, University of Ottawa, 200 Lees Avenue, Ottawa, ON K1N 6N5 Canada; 2 Centre of Vision Research & Department of Biology, York University, Toronto, ON M3J 1P3 Canada; 3 Department of Kinesiology, University of Waterloo, Waterloo, ON N2L 3G1 Canada

**Keywords:** Cohen’s kappa, Bodyweight bias, Movement screens, Motion and shape capture from sparse markers (MoSh)

## Abstract

**Background:**

The literature shows conflicting results regarding inter- and intra-rater reliability, even for the same movement screen. The purpose of this study was to assess inter- and intra-rater reliability of movement scores within and between sessions of expert assessors and the effects of body-shape on reliability during a movement screen using a custom online visualisation software.

**Methods:**

Kinematic data from 542 athletes performing seven movement tasks were used to create animations (i.e., avatar representations) using motion and shape capture from sparse markers (MoSh). For each task, assessors viewed a total of 90 animations. Using a custom developed visualisation tool, expert assessors completed two identical sessions where they rated each animation on a scale of 1–10. The arithmetic mean of weighted Cohen’s kappa for each task and day were calculated to test reliability.

**Results:**

Across tasks, inter-rater reliability ranged from slight to fair agreement and intra-rater reliability had slightly better reliability with slight to moderate agreement. When looking at the average kappa values, intra-rater reliability within session with and without body manipulation and between sessions were 0.45, 0.37, and 0.35, respectively.

**Conclusions:**

Based on these results, supplementary or alternative methods should be explored and are likely required to increase scoring objectivity and reliability even within expert assessors. To help future research and practitioners, the custom visualisation software has been made available to the public.

## Background

Movement screens are used across a variety of ergonomic, clinical, and athletic settings to quantify ‘movement quality’ and identify movement patterns that are associated with an increased risk of injury and/or decreased performance [1–6]. There are many different types of movement screens, with the Functional Movement Screen (FMS) being the most well-known [6]. Each screen has its own unique battery of movements and scoring criteria, but each share one constant: they are all scored using visual appraisal, which is a subjective approach [6].

Within the literature, there are conflicting results regarding inter- and intra-rater reliability, even within the same movement screen [6]. When looking across studies, this is most likely attributed to the small number of raters being compared within each study (with the majority of studies using only two raters), rater experience, real-time scoring versus scoring from videos, and the qualitative interpretation of reliability measures [6]. When looking within studies, the variability in inter- and intra-rater reliability is thought to be due to the dynamic nature of the movements, the rater’s perspective, and/or rater bias [7, 8].

For movement screens, the movements can be dynamic and fast-paced in nature involving multiple joints, making it difficult for the rater to evaluate all parts of the movement across all of the joints [7]. In addition, the rater’s perspective may have an influence on the score, as they may only see the performance from one vantage point, making it difficult for the rater to see scoring criteria that are either out of view or occluded by the athlete’s body [7, 8]. To combat some limitations, preliminary evidence reports that scoring movements from video could increase the reliability [6], since assessors are able to watch the movement multiple times. However, a limitation of video is that the movement is reduced to one or a few vantage points, where important information may be out of view of the assessor. Although, to the best of the authors’ knowledge, weight-bias specifically during a movement screen has not been studied, research has consistently shown that there is pervasive implicit and explicit weight bias among clinicians, physical therapists, physical education teachers, and strength and conditioning personnel, with males showing a larger bias [9]. However, the ability to combine motion capture and animation techniques, may help correct potential biases. For example, by animating a generic shaped avatar using subject-specific motion data, a clinician could view the motion from different perspectives by changing the camera view of the animation or play the motion multiple times. In addition, because the subject’s look and shape are replaced by a generic avatar, the potential for body shape to bias scoring is removed. We developed a custom visualisation tool that allows subject-specific motions to be animated by using generic avatars by leveraging a technique known as motion and shape capture from sparse markers (MoSh) [10].

MoSh is an animation technique that translates 3D kinematic optical motion capture data into 3D animations visualizing both the kinematic movement patterns and the body-shape of the individual [10, 11]. With MoSh, there are three body-shape model templates (male, female, non-binary), that are manipulated by 10 weights that determine the contribution of blendshapes representing eigenshapes to fit a personalized body-shape to each individual [11]. Additional blendshapes model pose-dependent changes in body shape – one for each degree of freedom in each of the body joints. When using MoSh and a custom developed visualisation tool, a shape model is animated, much like an avatar in a video game, where the animation can be replayed multiple times, multiple raters can score the same movement, and the vantage point can be rotated to focus on specific points of interest. While 2-dimensional video can also be replayed and assessed by multiple raters, a key benefit of MoSh is that shape models can be manipulated and rigged to different kinematic movement patterns which allows for the effect of body-shape on reliability to be studied by creating animations with identical movement patterns, but differing body-shapes.

Therefore, the purpose of this study was to assess the inter- and intra-rater reliability between expert raters during a movement screen without strict scoring criteria using a custom developed visualisation tool with MoSh animations. For intra-rater reliability, reliability between two sessions and within the same session with and without body-shape modification was assessed. It was hypothesized that the intra-rater reliability within the same session would have the best reliability, followed by the intra-rater reliability between sessions without body-shape modification, and subsequently intra-rater reliability within sessions with body-shape modification and inter-rater reliability.

## Methods

### Study design and ethical approval

The study, where the authors aimed to investigate inter-rater and intra-rater reliability in movement assessments, specifically examining the influence of athletes’ body shapes on these evaluations, was approved by the research ethics board at the University of Ottawa (*file no: H-10-19-4983*). Experts in orthopedics, physiotherapy, strength and conditioning, kinesiology, and movement performance were recruited as raters for this study. The study utilized motion capture data from 542 athletes to create 630 animations showcasing seven different common screening movements. This approach allowed for a detailed examination of reliability across and within sessions and the exploration of potential weight biases in assessments. Ethical considerations were addressed through informed consent procedures before and after the study, with participants initially unaware of the investigation’s full scope to minimize bias.

### Settings and participants

Raters with expertise in orthopedics, physiotherapy, strength and conditioning, kinesiology, and movement performance were recruited for this study. Before data collection started, each rater was asked to fill out an online form providing demographic information including age, gender, job title, years of experience, certifications, and average number of movement assessments performed per day, week, month or year. The consent form outlined the purpose of the study as to examine the inter-rater reliability of the used dataset. To try and obtain unbiased and/or natural reactions, the purposes of examining intra-rater reliability between sessions and within sessions were omitted.

### Procedures

#### Animation preparation

**Fig. 1:**
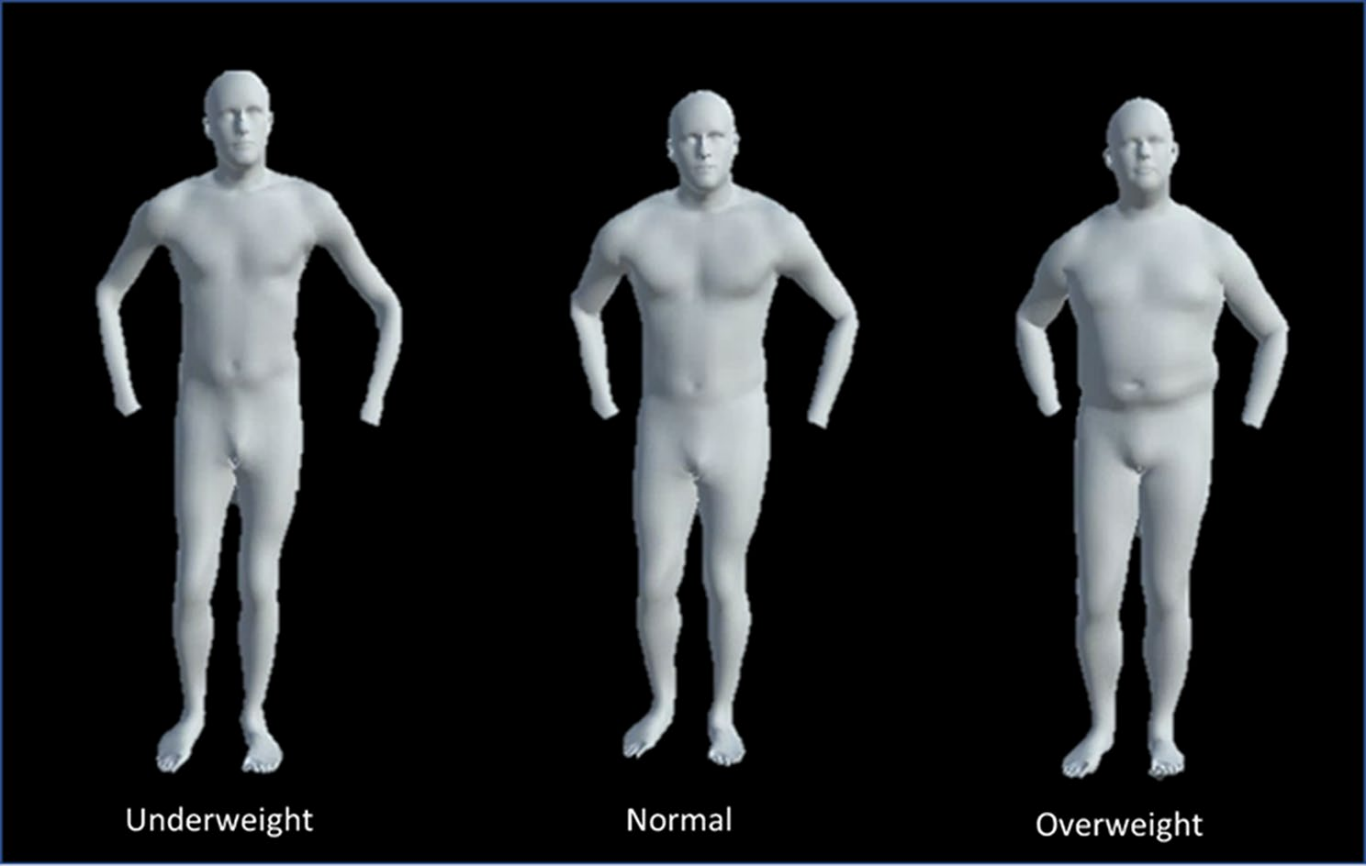
An example of the three different body-shapes (underweight, normal, and overweight) used for the intra-rater reliability within session with body-shape modification

To create the animations, motion capture data from 542 athletes (473 males, 69 females) performing seven unique movement screening movements (i.e., bird-dog, drop-jump, hop-down, L-hop, lunge, step-down, and T-balance) were collected in the USA between 2012 and 2016. At the time of collection, athletes competed in one of 12 sports (i.e., baseball, basketball, cricket, football, golf, lacrosse, rugby, soccer, squash, tennis, track and field, or volleyball) and ranged in skill level from youth to professional (e.g., NFL, NBA, MLB, FIFA). The average age, height, weight were 20.2 ± 4.7 years, 183.3 ± 19.3 cm, and 83.1 ± 22.9 kg, respectively. Athletes were included in the study as long as they were physically able to compete in practices and games at the time of collection. To collect whole body kinematics, 42 markers were placed on anatomical landmarks and captured using an 8-camera Raptor-E motion capture system (Motion Analysis, Santa Rosa, CA, USA). All data were labelled and gap-filled in Cortex (Motion Analysis, Santa Rosa, CA, USA). Once the data were cleaned, MoSh was applied to the data. For MoSh, body-shape and kinematic data are coded so they can be manipulated independently from one another. Body-shape is able to be manipulated by adjusting the 10 weights that represent body-shape, whereas kinematic data can be altered by changing joint angles and how they change over time. The marker set used, while resembling the ideal marker set proposed by Loper et al., 2014, was not identical. Differences included the absence of markers positioned on the breasts, buttocks, and hands. The breast and buttock markers were pertinent for fitting the female body-shape model; therefore, only male motion data were retained for this analysis. The hand markers were necessary to create realistic hand movements. Since our data did not include hand markers, we removed the hands from the animations. For this study, the 5th, 50th, and 95th percentile body-mass indexes (BMI) of the dataset were calculated and used as the cut-offs for the three body-shape classes: underweight, normal, and overweight (Fig. 1).

**Fig. 2:**
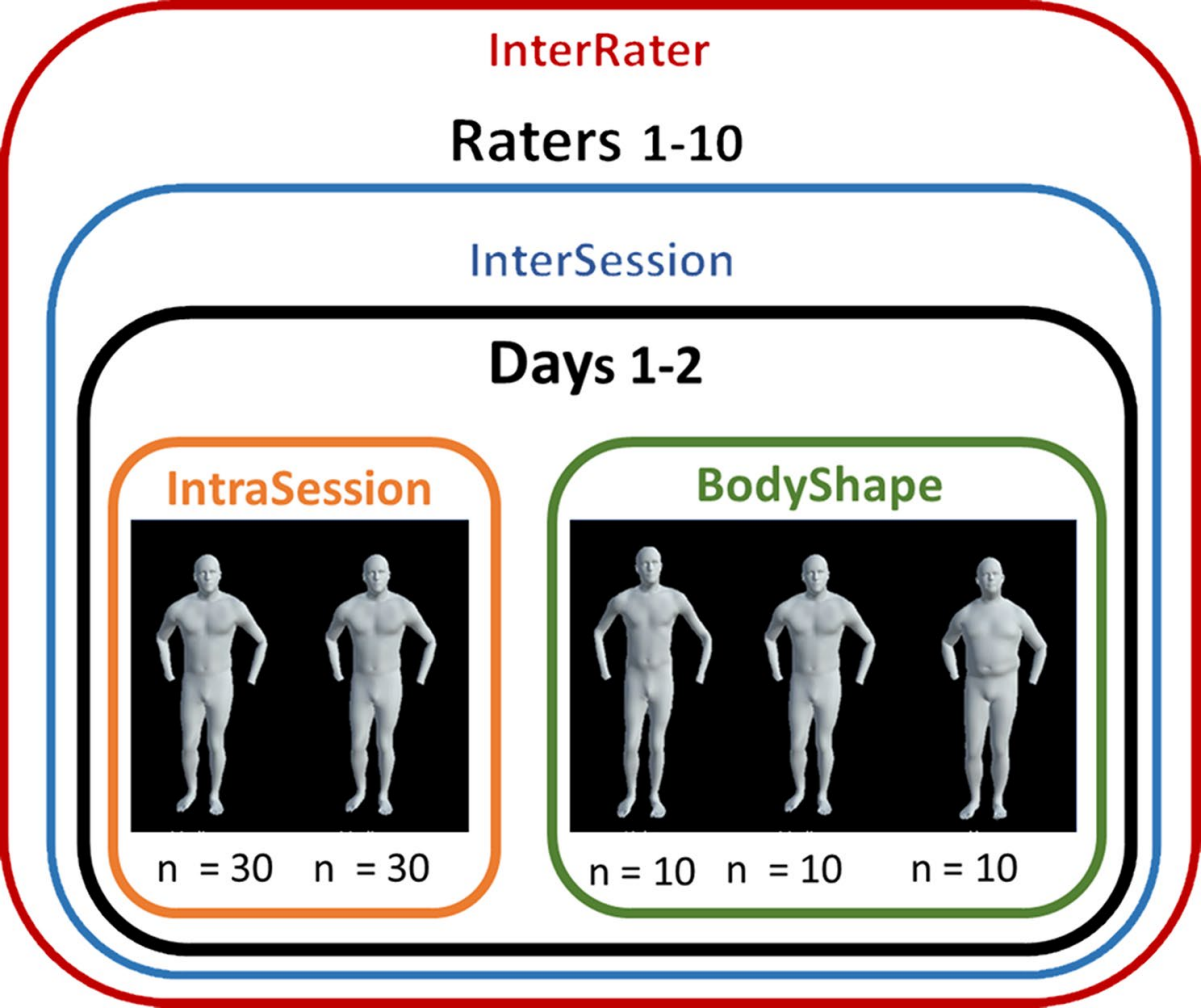
A visual depiction of the animations being compared to assess inter- and intra-rater reliability. InterRater = inter-rater reliability between raters. InterSession = intra-rater reliability between days. IntraSession = intra-rater reliability within session without body-shape modification. BodyShape = intra-rater reliability within session with body-shape modification

A database of 630 animations was created consisting of 90 animations from each of the seven movements (7 movements x 90 animations = 630 animations). For each of the seven movement tasks, animations were created to be able to test for intrasession reliability, intersession reliability, and weight bias (Fig. 2), as well as having a diversity of movement competency levels with approximated scores ranging from 1 to 10 with 10 being the best, which were selected based on scoring from two pilot raters. Two pilot raters assessed the animations without specific scoring criteria. Movement profiles were considered only when there was agreement between the raters. Due to criticism of scoring criteria for lacking sensitivity, we opted for a 0–10 scale to enhance sensitivity in our evaluations. Subsequently, animations with diverse movement scores between 1 and 10, reflecting the raters’ assessments, were chosen as the 30 movers, with 10 of them selected for body-shape manipulation. To test intrasession reliability, 30 different movers with unique movement patterns and body-shape were generated and duplicated, to create 60 of the 90 animations (Fig. 2). In the debrief, after revealing the body-shape manipulation, some raters disclosed their biases. Interestingly, some found it easier to rate individuals with more wobbly mass, citing it as an indicator of stability. Others found it challenging, as they believed the wobbly mass motion detracted from the underlying motion pattern. To test weight-bias, for each approximated score, three animations were created with identical movement patterns but body-shape was manipulated so each of the three animations had a body-shape of a different class (e.g., underweight, normal, overweight), making up the remaining 30 animations (10 movement scores x 3 weight classes; Fig. 2). If a movement task was performed bilaterally, only animations for the right-side were included.

### Software preparation

**Fig. 3:**
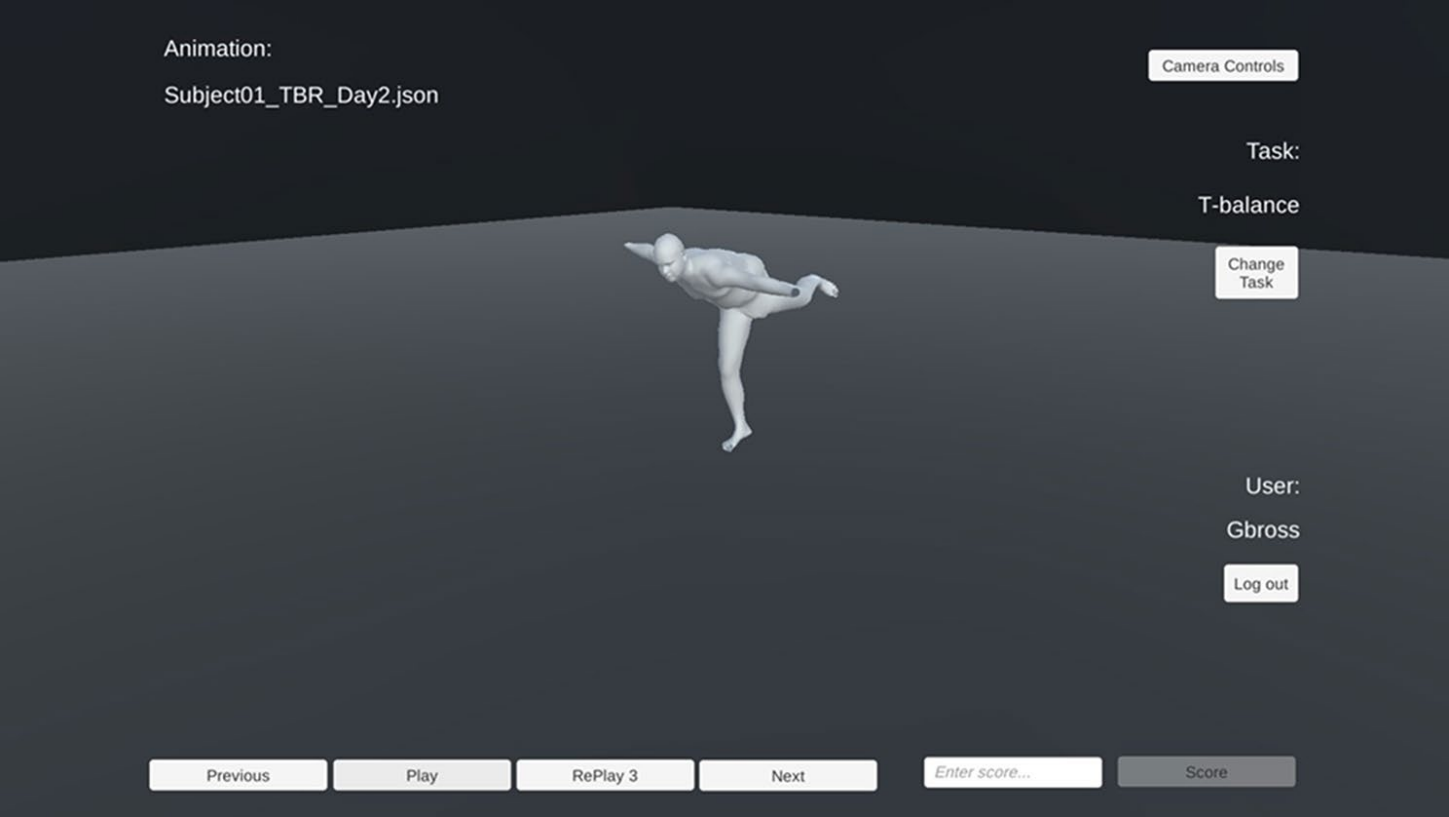
A screenshot of the custom visualisation tool user interface

A custom-built, online, visualisation software was developed using the Unity game engine (Unity Software Inc., San Francisco, CA, USA), which was deployed on a Compute Canada server and linked to a common domain name. Within the software, there were three modules: Training, Day 1, and Day 2. Within each module, the raters were able to: zoom, rotate, and translate the animation for 360° views; play the animation; replay the animation; score the animation; move between the next and previous animation; view the control short-cut keys; and return to the main menu (Fig. 3). For each animation, the score, date and time of score, time to score, and number of replays were recorded and stored in a MariaDB database using phpMyAdmin.

### Protocol and outcome measures

The study consisted of three modules: Training, Day 1 and Day 2. Before beginning to score movements, raters partook in the training module, where five animations for each movement were at their disposal to study. To select training module animations, two pilot raters completed Day 1 of the protocol and animations were chosen that had complete agreement between the two raters. Since the training module animations were part of the testing database, depending on the movement task, the training animations either had a score of {1, 3, 5, 7, or 9} or {2, 4, 6, 8, or 10}, to minimize the number of animations the raters were exposed to prior to the start of the study. In order to minimize bias, the pilot raters’ scores were shown, but explanations for each score were not provided. Raters were asked to use their training and expertise to determine their own scoring criteria based on whole-body kinematics of the given training animations. The raters were able to return to the training module at any time during the study and were able to replay the animations as many times as they liked.

For the Day 1 and Day 2 module, raters scored each animation from 1 to 10 based on the animation’s movement competency for each movement task with 10 being the best. In order to decrease the risk of fatigue, raters did not have to complete all modules in one sitting but were able to complete them at their own pace. In addition, the raters were able to score the movements in whichever order they chose. The Day 1 and Day 2 modules had identical animations; however, the order in which the animations were presented within each task were different between the two days. To decrease the risk of a learning effect, raters had to wait a minimum of 48 h after completing the Day 1 module of the movement task before starting the Day 2 module of the same movement task. Raters were only able to replay each movement three times at real-time speed, but had the ability to zoom, translate, and rotate the vantage point during the movement. The limited number of replays was to decrease the risk of recall bias, especially since many of the movements were duplicates. If a rater submitted multiple scores for the same animation, only the last score was registered. After completing the Day 1 and Day 2 module, the true purposes of the study were disclosed, and the raters signed a post-study consent form that confirmed their acknowledgment and understanding of the use of deception in the study and permission to use their data. All participants completed both modules, except for one female who only completed Day 1.

### Data analysis

To test inter- and intra-rater reliability, the arithmetic means of weighted Cohen’s kappa were used. For inter-rater reliability, comparisons between each rater and the mean of the 44 weighted Cohen’s kappa values were calculated for each movement task. For the intra-rater reliability between sessions, weighted Cohen’s kappa was calculated for each rater (except Rater 3 who only completed Day 1) between the exact same movements for Day 1 and Day 2. Both the individual and mean kappa values were retained for each movement task. For intra-rater reliability within session without body-shape manipulation, weighted Cohen’s kappa was calculated between the 30 unique movements for each rater for each day, resulting in 19 kappa values (10 raters for Day 1 + 9 raters for Day 2) for each movement task. To investigate intra-rater reliability within session when body-shape was manipulated, weighted Cohen’s kappa between each weight class for the 10 unique movements per day was calculated resulting in three kappa values (Overweight-Normal, Overweight-Underweight, Normal-Underweight) per rater per day. The kappa values were then averaged within raters. Weighted Cohen’s kappa values were interpreted as no (≤ 0), slight (0.01–0.20), fair (0.21–0.40), moderate (0.41–0.60), substantial (0.61–0.80), and almost perfect (0.81-1.00) agreement [12].

## Results

In this study, ten expert movement assessors (6 males, 4 females) evaluated motion capture data from 542 athletes (473 males, 69 females) across 12 sports—ranging from baseball to volleyball—with skill levels from youth to professional (e.g., NFL, NBA). These athletes, averaging 20.2 years old, performed seven distinct movements such as bird-dog and lunge. The assessors, including orthopedic surgeons and physical therapists among others, had an average of 7 years of experience and regularly conducted movement assessments in their professional roles.

For inter-rater reliability, there was slight to fair agreement with kappa values ranging from 0.09 (bird-dog) to 0.33 (lunge) across all movement tasks (Table 1). For intra-rater reliability between sessions, across raters, there was fair to moderate agreement with kappa values ranging from 0.27 (L-hop) to 0.46 (step-down; Table 2). For intra-rater reliability within session without body-shape manipulation, there was fair to moderate agreement with kappa values ranging from 0.33 (step-down) to 0.58 (T-balance) across all tasks (Table 3). For intra-rater reliability within session with body-shape manipulation, there was slight to moderate agreement with kappa values ranging from 0.17 (drop-jump) to 0.52 (Lunge) across all tasks (Table 4).

**Table 1:** The arithmetic mean of weighted Cohen’s kappa across all raters and days for inter-rater reliability, intra-rater reliability between sessions (InterSessions), intra-rater reliability within sessions without body-shape modification (IntraSession), and intra-rater reliability within session with body-shape modifications (BodyShape) for each task and the average and standard deviation across tasks

	BD		DJ		HD		LH		LG		SD		TB		Average	STD
	Day 1	Day 2	Day 1	Day 2	Day 1	Day 2	Day 1	Day 2	Day 1	Day 2	Day 1	Day 2	Day 1	Day 2		
InterRater	0.09	0.10	0.14	0.14	0.13	0.12	0.14	0.11	0.31	0.33	0.21	0.25	0.22	0.18	0.18	0.08
Between Sessions	0.51		0.32		0.23		0.14		0.42		0.48		0.37		0.35	0.13
Within Session	0.47	0.46	0.43	0.48	0.41	0.35	0.36	0.46	0.48	0.51	0.33	0.39	0.54	0.58	0.45	0.07
BodyShape	0.41	0.41	0.17	0.26	0.29	0.29	0.39	0.50	0.52	0.46	0.34	0.35	0.40	0.35	0.37	0.10

*BD = bird-dog, DJ = drop-jump, HD = hop-down, LH = L-hop, LG = lunge, SD = step-down, and TB = T-balance

**Table 2:** The weighted Cohen’s kappa for intra-rater reliability between sessions for each rater for each task and the average and standard deviation (STD) across tasks and across raters

Subject	BD	DJ	HD	LH	LG	SD	TB	Average		STD
1	0.35	0.29	0.23	0.25	0.32	0.43	0.44	0.33	0.08	
2	0.03	0.27	0.23	0.16	0.31	0.20	0.36	0.22	0.11	
3	n/a	n/a	n/a	n/a	n/a	n/a	n/a	n/a	n/a	
4	0.51	0.24	0.30	0.32	0.56	0.67	0.36	0.43	0.16	
5	0.47	0.48	0.43	0.30	0.57	0.61	0.63	0.50	0.12	
6	0.38	0.37	0.43	0.54	0.52	0.39	0.64	0.47	0.10	
7	0.28	0.19	0.29	0.20	0.40	0.33	0.36	0.29	0.08	
8	0.51	0.32	0.23	0.14	0.42	0.48	0.37	0.35	0.13	
9	0.45	0.30	0.33	0.35	0.52	0.58	0.46	0.43	0.10	
10	0.35	0.49	0.21	0.20	0.44	0.43	0.47	0.37	0.12	
**Average**	0.37	0.33	0.30	0.27	0.45	0.46	0.45	0.38	n/a	
**STD**	0.15	0.10	0.09	0.13	0.10	0.15	0.11	n/a	0.14	

*BD = bird-dog, DJ = drop-jump, HD = hop-down, LH = L-hop, LG = lunge, SD = step-down, and TB = T-balance

**Table 3:** The arithmetic mean of weighted Cohen’s kappa for intra-rater reliability within session without body-shape modification for each rater for each task and session day, as well as, the average and standard deviation (STD) across raters and tasks

Rater	BDR					DJ				HDR				LHR				LG				SDR				TBR				Average			STD		
	Day 1			Day 2		Day 1		Day 2		Day 1		Day 2		Day 1		Day 2		Day 1		Day 2		Day 1		Day 2		Day 1		Day 2		Day 1	Day 2		Day 1		Day 2
1	0.48			0.42		0.62		0.45		0.37		0.33		0.39		0.54		0.32		0.64		0.51		0.43		0.52		0.60		0.46	0.49		0.11		0.11
2	0.49			0.27		0.26		0.31		0.30		0.12		0.08		0.40		0.36		0.28		0.22		0.48		0.45		0.41		0.31	0.32		0.14		0.12
3	0.41			n/a		0.60		n/a		0.26		n/a		0.34		n/a		0.53		n/a		0.25		n/a		0.47		n/a		0.41	n/a		0.13		n/a
4	0.51			0.46		0.36		0.51		0.59		0.41		0.32		0.60		0.45		0.47		0.43		0.31		0.50		0.56		0.45	0.48		0.09		0.10
5	0.34			0.52		0.47		0.58		0.44		0.43		0.37		0.50		0.60		0.52		0.39		0.43		0.59		0.66		0.46	0.52		0.10		0.08
6	0.52			0.54		0.61		0.42		0.56		0.43		0.55		0.68		0.69		0.49		0.32		0.60		0.74		0.72		0.57	0.55		0.14		0.12
7	0.50			0.42		0.33		0.50		0.32		0.34		0.21		0.40		0.34		0.35		0.02		0.30		0.58		0.47		0.33	0.40		0.18		0.07
8	0.55			0.49		0.17		0.51		0.40		0.32		0.36		0.09		0.43		0.59		0.35		0.34		0.46		0.58		0.39	0.42		0.12		0.18
9	0.40			0.49		0.37		0.47		0.35		0.39		0.50		0.41		0.53		0.72		0.50		0.41		0.51		0.54		0.45	0.49		0.07		0.11
10	0.49			0.57		0.54		0.56		0.52		0.42		0.49		0.53		0.52		0.50		0.33		0.18		0.52		0.65		0.49	0.49		0.07		0.15
**Average**	0.47			0.46		0.43		0.48		0.41		0.35		0.36		0.46		0.48		0.51		0.33		0.39		0.54		0.58		0.45			n/a		n/a
**STD**	0.06			0.09		0.16		0.08		0.11		0.10		0.14		0.17		0.12		0.14		0.14		0.12		0.09		0.10		n/a	n/a		0.13		

*BD = bird-dog, DJ = drop-jump, HD = hop-down, LH = L-hop, LG = lunge, SD = step-down, and TB = T-balance

**Table 4:** The arithmetic mean of weighted Cohen’s kappa for intra-rater reliability within session with body-shape modification for each rater for each task and session day, as well as, the average and standard deviation (STD) across raters and tasks

Rater	BDR		DJ		HDR		LHR		LG		SDR		TBR		Average		STD	
	Day 1	Day 2	Day 1	Day 2	Day 1	Day 2	Day 1	Day 2	Day 1	Day 2	Day 1	Day 2	Day 1	Day 2	Day 1	Day 2	Day 1	Day 2
1	0.51	0.38	0.69	0.23	0.46	0.31	0.49	0.65	0.47	0.37	0.48	0.52	0.43	0.30	0.50	0.40	0.09	0.14
2	0.27	0.24	0.12	0.15	0.15	0.37	0.05	0.42	0.56	0.42	0.07	0.07	0.34	0.30	0.22	0.28	0.18	0.14
3	0.48	n/a	0.00	n/a	0.04	n/a	0.57	n/a	0.34	n/a	0.25	n/a	0.30	n/a	0.28	n/a	0.21	n/a
4	0.70	0.69	0.12	0.27	0.50	0.60	0.42	0.47	0.72	0.69	0.73	0.50	0.42	-0.10	0.52	0.45	0.22	0.28
5	0.35	0.36	0.18	0.25	0.13	0.39	0.48	0.34	0.55	0.61	0.47	0.33	0.42	0.53	0.37	0.40	0.16	0.12
6	0.14	0.53	0.09	0.06	0.63	0.51	0.55	0.66	0.58	0.79	0.16	0.62	0.41	0.60	0.37	0.54	0.23	0.23
7	0.19	0.25	0.15	0.37	0.05	-0.02	0.47	0.45	0.44	0.00	0.17	0.03	0.22	0.33	0.24	0.20	0.15	0.20
8	0.63	0.50	-0.03	0.16	0.21	0.16	0.45	0.60	0.58	0.35	0.47	0.50	0.45	0.28	0.40	0.36	0.23	0.18
9	0.46	0.39	0.13	0.20	0.38	0.23	0.28	0.26	0.43	0.37	0.16	0.10	0.38	0.45	0.32	0.29	0.13	0.12
10	0.36	0.33	0.20	0.64	0.33	0.07	0.15	0.65	0.56	0.54	0.42	0.51	0.63	0.45	0.38	0.46	0.18	0.20
**Average**	0.41	0.41	0.17	0.26	0.29	0.29	0.39	0.50	0.52	0.46	0.34	0.35	0.40	0.35	0.37		n/a	n/a
**STD**	0.18	0.15	0.20	0.17	0.20	0.20	0.18	0.15	0.10	0.23	0.21	0.23	0.11	0.20	n/a	n/a	0.20	

*BD = bird-dog, DJ = drop-jump, HD = hop-down, LH = L-hop, LG = lunge, SD = step-down, and TB = T-balance

When looking at the individual rater level, for intra-rater reliability between sessions averaged across movement tasks, raters had fair to moderate agreement, with kappa values ranging from 0.22 (Rater 2) to 0.50 (Rater 5; Table 2). For intra-rater reliability within sessions without body-shape modification, reliability ranged from fair to moderate reliability with kappa values ranging from 0.31 (Rater 2, Day 1) to 0.57 (Rater 6, Day 1; Table 3). For intra-rater reliability within session with body-shape modification, reliability ranged from slight to moderate agreement with kappa values ranging from 0.2 (Rater 7, Day 2) − 0.54 (Rater 6, Day 2; Table 4).

## Discussion

The purpose of this study was to examine the inter-rater and intra-rater reliability of movement competency scores during a movement screen between and within sessions using a customized visualisation tool and to assess the effects of body-shape on reliability. Our findings indicate that intra-rater reliability within the same session without body-shape manipulation showed the highest reliability, followed by intra-rater reliability between sessions and intra-rater reliability within sessions with body-shape manipulation, with inter-rater reliability demonstrating the lowest agreement. These results suggest a trend where reliability diminishes as the complexity of the scoring situation increases, specifically when body shape is altered between sessions. Our analysis revealed that both forms of intra-rater reliability ranged from slight to moderate, and inter-rater reliability varied from slight to fair across different movement tasks and raters. The kappa values observed were relatively low, which aligns with previous studies where intra-rater reliability generally surpasses inter-rater reliability due to consistent personal bias and scoring perspectives maintained by individual raters over time. Notably, these findings are consistent with previous research that suggests greater scoring range and rater number can reduce reliability due to increased scoring complexity and variability in rater perception and criteria.

Compared to other studies, the kappa values were on the lower end of the spectrum; however, the pattern of intra-rater reliability being better than inter-rater reliability was similar to previous results [7, 8, 13]. The lower scores could be due to the larger number of possible scores, the greater number of raters being compared, or the difference in scoring criteria. The FMS is scored between 0 and 3 for each task [2, 14, 15], whereas the movements for this study were scored between 1 and 10. With the greater number of possible scores, there is greater sensitivity; however, the probability of raters selecting the same score is decreased. In addition, the sensitivity may be greater, but the human eye may not be able to distinguish the differences. Previous studies compared 2 [7], 3 [13] and 4 [8] raters, whereas this study compared 10 raters. The increase in number of raters, due to needing to align more raters, may also contribute to the lower kappa values.

Although the greater range in scores and number of raters likely contributed to the lower kappa values, the main reason was likely due to the scoring criteria. For the movement screens that previously assessed inter-rater and intra-rater reliability, strict task-specific scoring criteria were used to assess movement competency [7, 8, 13], whereas for this study, the raters were asked to use their expertise to establish their own whole-body scoring criteria. Previous research has criticized the FMS for having poor criterion validity, which was attributed to the vagueness of the scoring criteria [16]. In addition, many of the FMS task-specific scoring criteria are not linked (epidemiologically or biomechanically) to injury mechanisms or risk factors [17] and individuals were able to increase their scores when made aware of the scoring criteria [18]. Furthermore, due to the large amount of movement variability between athletes, the FMS scoring criteria may be insensitive to potentially risky movement behavior, with previous research recommending that whole-body segment and joint kinematics should be incorporated when administering movement screens [17]. Therefore, for this study we opted to test the reliability of movement competency scores during the movement screen with less specific scoring criteria, which likely led to the lower reliability scores compared to previous research.

For intra-rater reliability, as hypothesized, the within session without body-shape modification had the highest reliability compared to within session with body-shape modification and between session reliability. The within session without body-shape modification were identical movements and avatars, which the raters would have seen sometimes only two animations previously, therefore since there was a shorter duration between rescoring the two movements compared to the between session, which had a minimum of 48 h between rescoring the animations, it was expected that the within session intra-rater reliability would be higher than the between session reliability. Similar results have been reported when comparing intra-rater reliability within and between session for the Soccer Injury Movement Screen [19]. The poor intra-rater reliability of movement scores when scoring identical animations suggests that visual observation is not precise when observing whole-body kinematics. This may be due to the large amount of information the rater needs to observe, process, and analyze the movements in a short amount of time [7]. In addition, due to the raters scoring multiple animations at once, the low intra-rater reliability may be because of a bias due to the influence of previously seen animations, where the bias may change with every new animation seen.

For the intra-rater reliability within session with body-shape modification, when averaging results within tasks across raters, the reliability was worse than the intra-rater reliability within session without body-shape modification. The without body-shape modification animations had identical movements and avatars, whereas, for the with body-shape modification the avatars looked different, which may contribute to the lower reliability. In addition, research has consistently shown that there is pervasive implicit and explicit weight bias among clinicians, physical therapists, physical education teachers, and strength and conditioning personnel [9].

When looking across raters, differences in average kappa values between without body-shape modification and with body-shape modification ranged from − 0.02 to 0.17, with a negative value indicating better agreement with body-shape manipulation. The single rater who had a slight increase in reliability with body-shape manipulation also had just over double the amount of variability in scores across tasks compared to the without body-shape modification condition, suggesting that the observed differences were most likely attributed to the large amount of variability seen across all conditions. The range in differences in kappa values between the two conditions suggests that some raters were more affected by body-shape than others. These differences are likely due to rater bias, which has been well documented with research suggesting that the rater bias can account for just as much variance of scores as differences in the examinee’s ability [20]. Biases can be conscious or unconscious with common types of biases including: leniency bias (inflating scores due to feeling sympathetic towards the ratee), contrast bias (evaluating by comparing to previous person), central tendency bias (preferring to give an average, middle rating despite performance), similar to me bias (inflating scores based on rater feeling similar to ratee), personal bias (scoring based on personal beliefs and ideologies), and halo effect (rating based only on one good aspect, despite the rest of the performance). Therefore, the variance in effect of body-shape on reliability between raters is likely due to differences in the type and severity of biases in effect. When looking at whether scores increased or decreased as BMI increased, there was no uniformed pattern. When talking with raters after disclosing the true purposes of the study, some raters acknowledged their own known biases, whereas others said that they found animations with higher BMIs easier to score based on being able to use wobbly mass as another source of information.

Two limitations of this study were the use of an identical dataset between Day 1 and Day 2 and the use of new software. The use of two identical datasets between Day 1 and Day 2 may have led to some learning effects. To try to combat the learning effects, raters had to wait a minimum of 48 h between finishing Day 1 and starting Day 2. When looking between days for the inter-rater reliability, on average, there was no difference in kappa values between Day 1 and Day 2. There was a very slight average decrease in kappa values from Day 1 and 2 for both intra-session reliability without body-shape manipulation and intra-session reliability with body-shape manipulation of 0.02. Based on these results, a learning effect does not appear to be influencing the results and differences seen at the individual or task level are more likely due to the large variability seen across all conditions. In addition, the use of a new software and the use of avatars rather than 2D video of human participants may have been influencing their scoring abilities. However, the software was intuitive to use, each rater had the ability to familiarize themselves with the software and controls in the training session before starting the testing sessions, raters could access the control descriptions during any point in testing, and anecdotally, no raters mentioned any difficulty of using the program. The researchers believe that the benefits that the program provided such as the ability to have 360° views of the athletes and the ability to modify body-shape outweighed the use of the software and avatars potentially minimally affecting the rater’s scores.

During the debriefing with the raters, following the revelation of body-shape manipulation, several raters voluntarily shared biases they had recognized in their own evaluations throughout their careers. Interestingly, opinions varied significantly: some raters mentioned that they found it easier to assess individuals with more wobbly-mass movement, as they could use the visible oscillations as indicators of stability. Conversely, other raters felt that this additional movement made it more difficult to accurately gauge the fundamental movement patterns of the athletes.

In summary, inter- and intra-rater reliability were low, with agreement ranging from slight to moderate, suggesting that assessing movement competency via subjective assessment is not reliable with non-task-specific scoring criteria. This is further compounded when athletes with different body-shape types are being assessed, with reliability decreasing on average across raters when body-shape was manipulated. This study supports previous literature which argues for the use and development of objective methods, tools, and thresholds to better assess movement competency [16, 17].

## Conclusions

Based on data from the current investigation, body-shape had a negative effect on reliability compared to without body-shape modification with differences potentially due to rater bias. With MoSh, one can manipulate personal characteristics of the animation, while maintaining movement patterns. Therefore, MoSh in combination with the customized visualisation software can provide a tool to minimize bias by being able to standardize personal characteristics which may be biasing raters’ scores such as: body-shape, facial expressions, gender expression, and race. Using a user interface, the tool is able to be easily customized based on number of movements, type of movements, scoring scale, and visual input type.

For both inter- and intra-rater reliability, at best for this study, there is fair reliability, which suggests that assessing movement competency via subjective assessment with non-task-specific scoring criteria is not a reliable method. However, as mentioned previously, the inclusion of specific scoring criteria has its own limitations, such as lack of sensitivity. Future work should focus on alternative scoring methods such as objective measurements and the development of data-driven thresholds to better assess movement competency [16, 17].

## Data Availability

The developed visualisation software is available on Github, https://github.com/Graham-Lab1/MovementScoring, with a full sample application with source code and documentation for future research or practice and can be modified to accommodate other virtual characters/environments or other animation/media file types (e.g., video).

## Abbreviations

MoSh: Motion and shape capture from sparse markers
FMS: Functional Movement Screen
BMI: Body-mass indexes
